# The Optimization of a T-Cell Resonator: Towards Highly Sensitive Photoacoustic Spectroscopy for Noninvasive Blood Glucose Detection

**DOI:** 10.3390/bios15040254

**Published:** 2025-04-16

**Authors:** Thasin Mohammad Zaman, Md Rejvi Kaysir, Shazzad Rassel, Dayan Ban

**Affiliations:** 1 Department of Electrical and Electronic Engineering (EEE), Khulna University of Engineering & Technology (KUET), Khulna 9203, Bangladesh; zaman1903069@stud.kuet.ac.bd; 2 Photonics Research Group, Department of EEE, Khulna University of Engineering & Technology (KUET), Khulna 9203, Bangladesh; 3 Department of Electrical and Computer Engineering, Tennessee State University, 3500 John A Merritt Bivs, Nashville, TN 37209, USA; srassel@tnstate.edu; 4 Department of Electrical and Computer Engineering, University of Waterloo, 200 University Ave. W, Waterloo, ON N2L 3G1, Canada; dban@uwaterloo.ca; 5 Waterloo Institute for Nanotechnology, University of Waterloo, 200 University Ave. W, Waterloo, ON N2L 3G1, Canada

**Keywords:** photoacoustic spectroscopy (PAS), acoustic resonator, T-cell, mid-infrared (MIR) spectroscopy, Q-factor, photoacoustic resonator (PAR)

## Abstract

Noninvasive blood glucose monitoring is crucial for diabetes management, and photoacoustic spectroscopy (PAS) offers a promising solution by detecting glucose levels through human skin. However, weak acoustic signals in PAS systems require optimized resonator designs for enhanced detection sensitivity. Designing such resonators physically is complex, requiring the precise identification of critical parameters before practical implementation. This study focused on optimizing a T-shaped photoacoustic resonator using finite element modeling in a COMSOL Multiphysics environment. By systematically varying the geometric design parameters of the T-cell resonator, a maximum increase in the pressure amplitude of 12.76 times with a quality factor (Q-factor) of 47.5 was achieved compared to the previously designed reference acoustic resonator. This study took a significant step forward by identifying key geometric parameters that influence resonator performance, paving the way for more sensitive and reliable noninvasive glucose monitoring systems.

## 1. Introduction

Photoacoustic spectroscopy (PAS) is a promising analytical technique for detecting chemical compositions by measuring the optical absorption properties of samples [1,2,3,4,5]. With its advantages of high sensitivity, specificity, and precision, PAS has found applications in diverse fields, including environmental monitoring [6], industrial safety, biomedical diagnostics [7], and especially gas sensing [8]. In the PAS process, laser radiation is generally used for the excitation of the vibrational states of molecules for noninvasive glucose sensing. For excitation sources, near-infrared (NIR) and mid-infrared (MIR) laser sources are generally used because of their strong glucose absorption properties in these spectral ranges. Among them, NIR measurements indicate relatively weak glucose absorption compared to MIR-based PAS due to background water absorption and a high penetration depth. The accuracy of these measurements is compromised by significant interference from tissue and other blood components with similar absorption spectra. Also, unlike MIR-based PAS measurements, NIR measurements are affected by strong tissue scattering [9,10]. This is why MIR-based PAS has gathered attention for its potential in noninvasive glucose monitoring by analyzing interstitial fluid (ISF) through the skin [11,12,13]. This approach addresses the growing need for the painless, accurate, and real-time monitoring of blood glucose levels in diabetic patients. Despite its advantages, the implementation of PAS for noninvasive glucose detection faces significant challenges, primarily due to the weak acoustic waves generated from the photo-thermal energy conversion process [14].

The sensitivity of a PAS system depends on several factors, including the intensity of the modulated incident light, the sensitivity of the microphone, and the shape and structural design parameters of the resonant cell [15]. Resonant cells, in particular, play a critical role in amplifying the photoacoustic signal and ensuring efficient photo-thermal–acoustic energy conversion [15]. The intensity of the PA signal is greatly increased by the resonator when the modulated frequency of the incident light matches the inherent frequency of the resonator [16]. So far, considerable research has focused on modifying the shape of photoacoustic cells. In 2014, M. A. Pleitez et al. [17] developed a closed T-type cell for noninvasive glucose monitoring. This cell features two cylindrical cavities (resonance and absorption) that are connected perpendicularly. Notably, the absorption cavity has an optical window at one end and is open to samples at the other end. In the same year, they also created a windowless resonant cell that is open at both ends [18]. In contrast to the previously mentioned closed cell, the open cell design helps minimize the effects of temperature fluctuations, changes in the air pressure, and the accumulation of humidity. As a result, the sensitivity and long-term stability of the cell are significantly enhanced. Consequently, open-type cells have drawn considerable attention in the field of biological sample detection [19,20,21,22]. For instance, J. Sim et al. [19,20] proposed a dominant resonance mode designed to match a microphone with an open cell. This method effectively improved the signal-to-noise ratio (SNR) and lowered the detection limit. Additionally, optimizing the size and shape of traditional H-type and T-type cells has led to the broader application of photoacoustic resonators (PARs) in trace gas detection [23,24,25,26,27]. For instance, X. Yin et al. developed a PA cell capable of detecting nitrogen dioxide (NO_2_) at sub-ppb levels with a large linear dynamic range [25]. In their studies, researchers have primarily focused on optimizing various PA cell shapes and achieving lower detection limits. However, these efforts have been limited by the absence of a systematic approach to achieving effective conceptual optimization designs.

In general, existing PAS systems often suffer from suboptimal resonator designs, limiting their ability to detect weak signals reliably. To address this limitation, considerable research has focused on optimizing the shape and structural parameters of PARs. In our recently published work [28], we summarized the different types of resonant cells used for noninvasive glucose detection through photoacoustic spectroscopy (PAS). We also highlighted key factors that need to be considered to enhance the system’s response. Notably, T-shaped resonators have been commonly used in most cases for their high sensitivity, high Q-factor, and improved signal-to-noise ratio, and numerical investigations have also been carried out to enhance their sensitivity [29]. In our recent work [30,31], we primarily designed a T-shaped resonator as a proof of concept for mid-infrared (MIR)-based photoacoustic spectroscopy (PAS), incorporating a single and dual-excitation wavelength quantum cascade laser-based system aimed at noninvasive glucose sensing. However, there has been no optimization work conducted on this initial PAR design. This acoustic resonator has the potential to amplify the generated signal in the acoustic domain. If the detected signal is amplified using a microphone without prior amplification in the acoustic domain, any noise present will also be amplified alongside the electrical signal, which is highly undesirable. Therefore, it is crucial to amplify the generated ultrasonic sound in the acoustic domain before converting it to an electrical signal. This approach helps extract accurate information carried by the signal. Consequently, the analysis and optimization of T-shaped resonators served as a key motivation for this research.

In this study, we focused on enhancing the amplitude of the signal at the resonant frequency using finite element modeling (COMSOL Multiphysics 6.2 software), while also evaluating the quality (Q) factor with a custom-developed MATLAB R2021a script. Furthermore, we propose an optimized T-cell resonator design aimed at improving the sensitivity of PAS for noninvasive glucose detection. Through computational simulations, we demonstrated that optimizations to various parameters in the geometry of the T-type resonator significantly enhanced the signal strength. Additionally, we identified a set of these parameters that provided an increase in the amplitude of the maximum pressure by 12.76 times compared to the reference amplitude. These findings pave the way for more accurate and reliable PAS for noninvasive glucose sensing.

## 2. General Principle of Using PARs for Signal Amplification and Simulation Method

### 2.1. Principle of PAS

**Figure 1:**
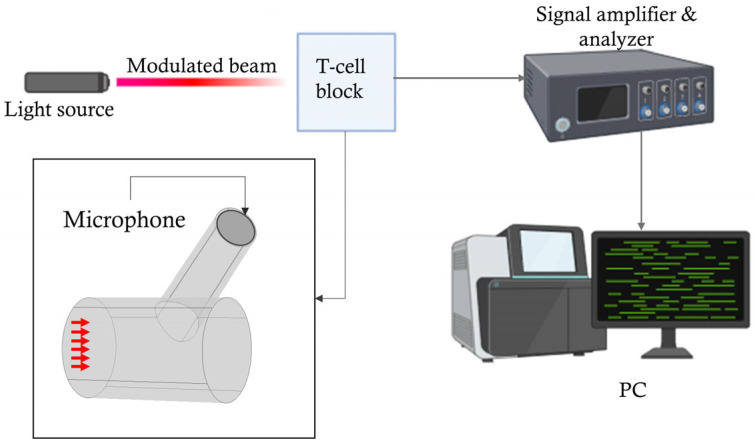
The basic experimental setup for generating and detecting acoustic signals in PAR-based photoacoustic spectroscopy (PAS). The detection arrangement includes a microphone (MIC), a lock-in amplifier, an analog-to-digital converter (A/D converter), a digital filter integrated into the signal amplifier and analyzer block, and a personal computer (PC).

Figure 1 shows a schematic view of the experimental setup used for the noninvasive detection of glucose concentrations using PAS. A modulated laser light penetrates the skin tissue, causing the generation of an acoustic signal. This acoustic signal is first amplified by a PAR in the acoustic domain and further enhanced by a lock-in amplifier for additional processing in the electric domain. In this study, we primarily focused on the design improvements and analysis of a PAR to achieve a higher output signal, which contributed to improved detection sensitivity.

#### Photoacoustic Resonator Model

**Figure 2:**
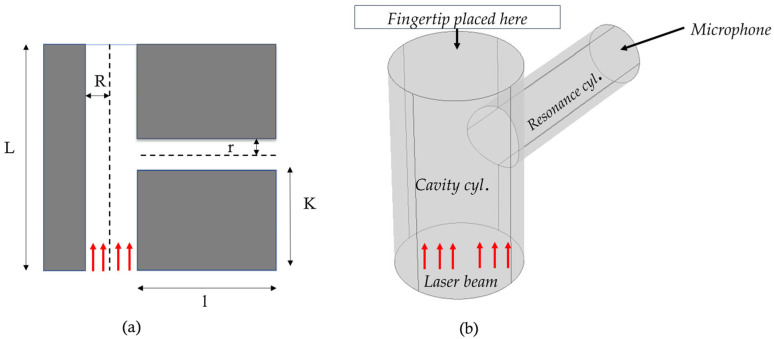
Schematic representation of T-shaped resonant cell structure with structural design parameters: (**a**) 2D cross-sectional view, (**b**) 3D view [here, cyl: cylinder].

Figure 2 presents a schematic representation of the PAR model. The dimensions and other parameters are described in the following table, Table 1. The red arrow indicates the direction of the incident laser beam. In practical applications, a human fingertip is positioned on top of the absorption cylinder. The modulated laser beam hits a portion of the fingertip and generates acoustic signals.

For simulation purposes, the cavity cylinder, or the specified domain, was coupled in series and driven by a harmonically oscillating pressure, as represented by Equation (1).

(1) $$p_{0}\left(t\right)=p_{0}e^{i\omega t}$$

(2) $$p_{0}=1+linper\left(1\right)$$

Here, $p_{0}\left(t\right)$ represents a harmonically oscillating pressure wave whose initial value is determined by $p_{0}$ and ω=2πf, where f denotes the frequency range we have given from 10 to 30 kHz. The function “linper()” in Equation (2) introduces a linear perturbation, which is a small variation in the initial pressure, $P_{0}\;$= “1 Pa”, value. Furthermore, the study settings provided a frequency range for ω. In this work, we set the frequency range from 8 to 32 kHz, as stated in the previous study [31].

### 2.2. Simulation Procedure

**Figure 3:**
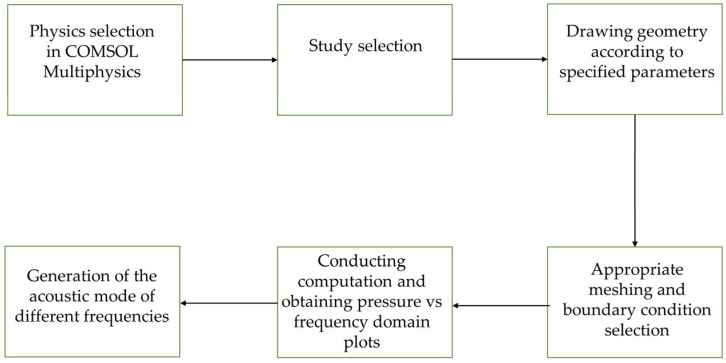
Flowchart of simulation procedures for modeling PAR.

Figure 3 illustrates the process of simulating resonator models, which began with selecting the appropriate physics. In this case, we used the pressure acoustic frequency domain (ACPR). Next, we chose the appropriate study methods, which included frequency sweep, frequency sweep with AWE (Asymptotic Waveform Evaluation), and modal solver frequency sweep methods. After that, we created the geometry, applied boundary conditions, and generated the mesh. We applied a sound-hard boundary wall condition. The boundary condition added a boundary at which the normal component of the acceleration, and thus the velocity, was zero [32]. This boundary wall helped to simulate a scenario where no signal could escape the enclosure. For meshing, we used a “user-controlled mesh”, setting the maximum and minimum element sizes to 0.924 mm and 0.00324 mm, respectively. Finally, we extracted the pressure versus frequency spectrum from the simulation, along with the various frequency modes identified as described in the following section.

The different types of study procedures are described as follows [33]:

- *The frequency domain solver* addressed problems subjected to harmonic excitation at specified excitation frequencies. It provided a numerically exact solution for each frequency, ensuring that the results were reliable, provided that the model converged and the meshing accurately resolved all relevant length scales in the physics.
- *The Asymptotic Waveform Evaluation (AWE)* did not compute the exact solution for every frequency. Instead, it performed a Taylor expansion of the solution around a few exact solutions, using lower-order approximations (such as Padé or Taylor) to estimate the solution across the desired frequency range.
- Additionally, *the Frequency Domain Modal* first determined a set of system eigenfrequencies and their corresponding eigenmodes, either by searching within a user-defined range or for a predetermined number of frequencies.

#### 2.2.1. Related Modeling Equations and Variables

This PAR model was designed to amplify the PA signal in COMSOL Multiphysics by using the “Pressure Acoustic frequency domain” module. For pressure acoustics, acoustic perturbations, or acoustic changes, to the ambient pressure are represented by the sound pressure *p*, which is obtained by solving for it using the pressure acoustics. The frequency domain, or time harmonic, formulation uses the inhomogeneous Helmholtz equation [34]:

(3) $$\nabla \left(\frac{1}{\rho_{c}}\left(\nabla p_{t}-q_{d}\right)\right)-\frac{{k_{eq}}^{2}p_{t}}{\rho_{c}}=Q_{m}$$

(4) $$p_{t}=p+p_{b}$$

(5) $${k_{eq}}^{2}={\left(\frac{\omega }{c_{c}}\right)}^{2}$$

The total pressure ($p_{t}$) is the sum of the input pressure (p), the possible background pressure field $(p_{b}$), $\rho_{c}\;$= *⍴* (density of air),$\;C_{c}$ = *c* (speed of sound), and $Q_{m}$, the monopole domain source. The boundary at which the normal component of the acceleration (and thus the speed) is zero is calculated as follows:

(6) $$-n\;.\left(-\frac{1}{\rho_{c}}\left(\nabla p_{t}-q_{d}\right)\right)=0$$

For a zero-dipole domain source ($q_{d}=0$) and constant fluid density, this means that the normal derivative of the pressure is zero at the boundary.

#### 2.2.2. COMSOL Terminal and Meshed View

**Figure 4:**
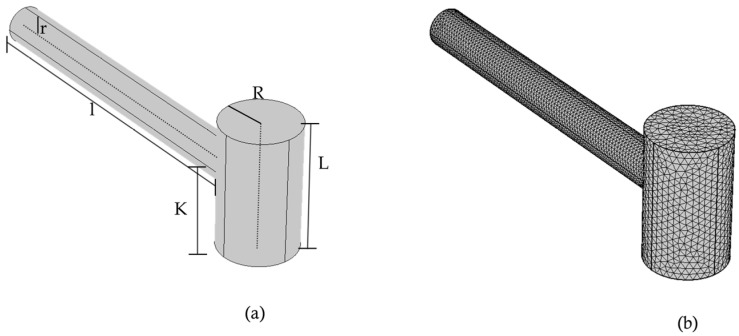
Pictorial representation of T-shaped resonator: (**a**) terminal view in COMSOL, (**b**) meshed representation.

Figure 4 presents a model of a PAR that requires a specific set of parameters for accurate simulation. These parameters include the resonance cylinder radius (*r*) and length (*l*), cavity cylinder radius (*R*) and length (*L*), sound speed (*C*), minimum frequency (min), and maximum frequency (max), among others. The values of these parameters were taken from Ref. [31] and used throughout the modeling process to simulate and compare the behavior of the resonator geometries. It is important to note that these parameters can be optimized to adjust the resonance frequency of the PAR for various applications.

#### 2.2.3. Quality (Q) Factor and Amplification Factor (α) Determination

A higher Q-factor indicates a sharper resonance peak and can be represented as follows:

(7) $$Q=\frac{f_{r}}{∆f}$$

where $f_{r}$ is the resonance frequency, while the increment *∆f* represents the difference in frequencies at which the pressure amplitude value has decreased to half of the resonance value. Improving the shape of the resonator can further optimize the amplification of the photoacoustic signal by enhancing the quality factor through a reduction in losses.

As the main objective of this work was to increase the acoustic signal (P) at a resonance frequency and compare it with the signal strength of the reference cell ($P_{ref})$, we defined a parameter amplification factor (α) as follows:

(8) $$\alpha =\frac{P}{P_{ref}}$$

## 3. Results and Discussion

### 3.1. Results Obtained Using Initial Parameters of Reference Cell

**Figure 5:**
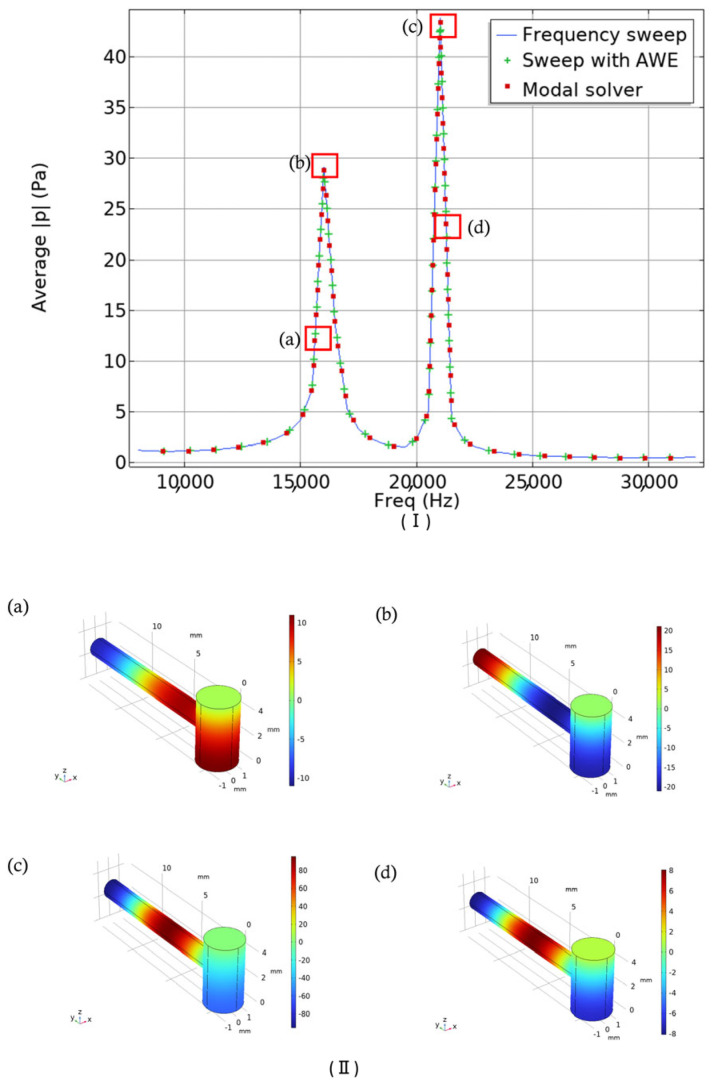
(**I**) Pressure versus frequency response of reference cell. (**II**) Corresponding mode representations of the T-shaped resonator (in Pa) as indicated by four points in Figure 5I, as indicated by red boxes.

In Figure 5I, the pressure versus frequency response is illustrated. A linear scale was used, and the frequency range was adjusted from 8 kHz to 32 kHz as in previous work [31]. In their experimental setup, they used a single-wavelength quantum cascade laser (QCL) with the laser current modulated from 10 kHz to 30 kHz. In Figure 5II, mode representations of the corresponding points in the previous figure are shown. In Figure 5IIa, the antinode (a point in a standing wave where the amplitude of the vibration is at its maximum) is positioned in the bottom portion of the cavity cylinder. For a resonator, placing a component such as the resonance cylinder at an antinode ensures maximum acoustic coupling, resulting in a stronger pressure signal. In Figure 5IIb, the antinode is perfectly located at the end of the resonant cylinder where the microphone will be placed. However, the other two mode representations show the antinode situated in the middle of the resonant cylinder. One of the main objectives of this work was to amplify the signal amplitudes identified in previous studies, which is why we selected this specific frequency range. In the upcoming section, we will primarily focus on the amplitude difference, which signifies the resonator’s ability to facilitate effective noninvasive blood glucose detection. Additionally, we will examine the quality factor and the effects of varying different parameters of our initially designed T-shaped cell.

The Q-factors associated with the designed model were 18.06 and 38.60 for the corresponding frequencies of 16 kHz and 21 kHz.

### 3.2. Effect of Changing Different Geometrical Parameters

#### 3.2.1. Effect of Changing Resonance Cylinder Parameters

**Figure 6:**
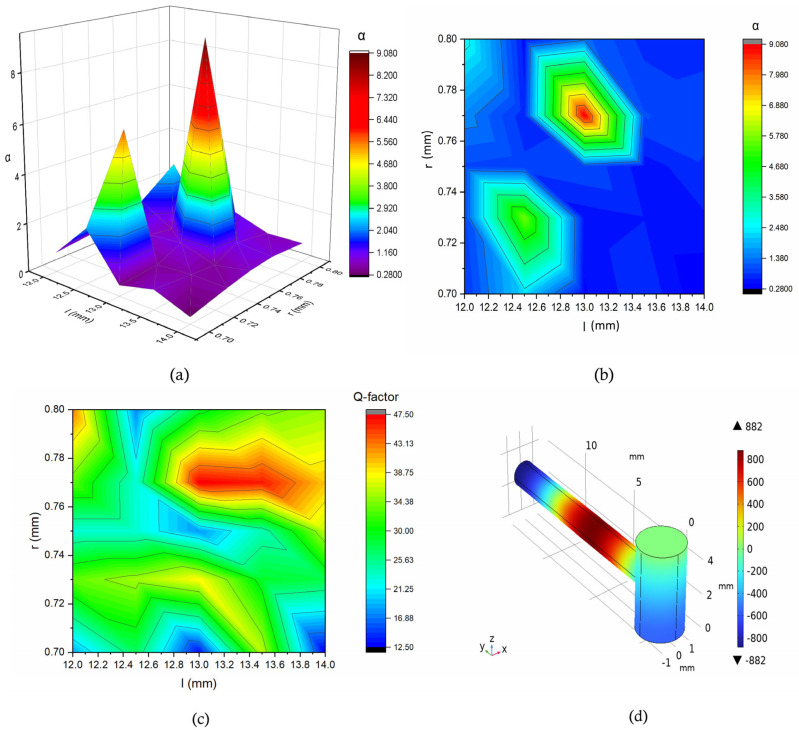
(**a**,**b**) Representation of the pressure amplification relative to the maximum pressure using a surface plot and contour plot for varying resonance cylinder parameters (r, l). (**c**) Contour plot of the Q-factor versus the resonance cylinder parameters. (**d**) Mode representation of the corresponding peak at a 21 kHz frequency in Pa, as indicated by the red points in the surface and contour plots in figures (**a**,**b**).

Figure 6 illustrates the effects of varying different geometric parameters of the resonance cylinder. In this context, we defined the ratio of the present value to the initial amplitude of the pressure as α. Our analysis indicated that for a specific combination of resonance cylinder parameters, we obtained a value of α equal to 9.07, indicating a significant amplification of the pressure values. By adjusting the parameters further, we achieved a greater amplification of the pressure versus frequency response. A contour plot was also generated to visualize the variation in the Q-factor as a function of *l* and *r*. The highest Q-factor (47.5) was observed at *l* = 13 mm and r = 0.77 mm, marked by a red region. The plot reveals a peak at around *l* = 13 mm, suggesting an optimal geometry for achieving the maximum resonance.

#### 3.2.2. Effect of Changing Cavity Cylinder Parameters

**Figure 7:**
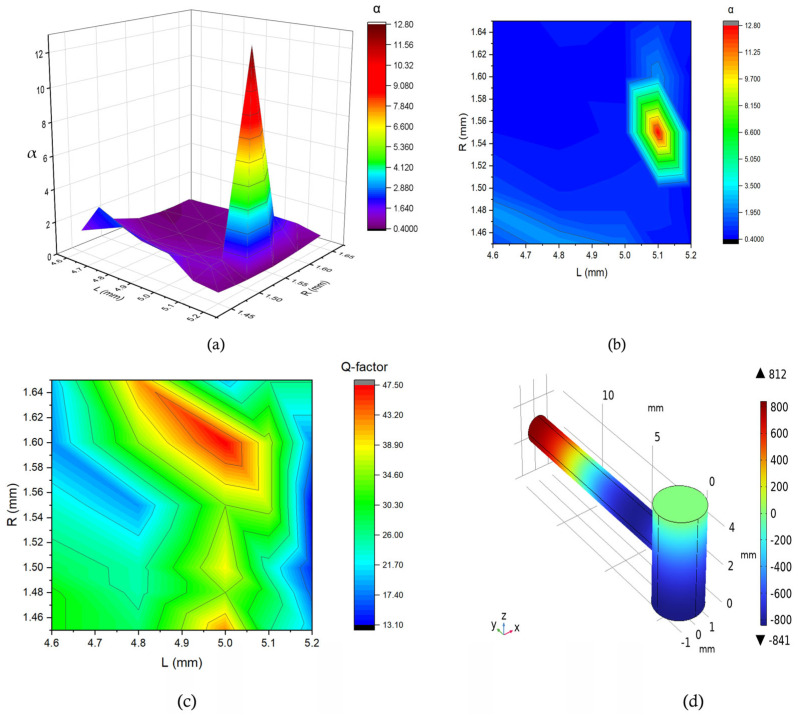
(**a**,**b**) Representation of the pressure amplification relative to the maximum pressure using a surface plot and contour plot for varying cavity cylinder parameters (R, L). (**c**) Contour plot of the Q-factor versus the cavity cylinder parameters. (**d**) Mode representation of the corresponding peak at a 16 kHz frequency in Pa, as indicated by the red points in the surface and contour plots in figures (**a**,**b**).

From Figure 7, we can observe that the value of α was 12.76, indicating a significant amplification of the initially generated pressure. In this case, for a combination of the values of *R* and *L*, which were 1.6 mm and 5 mm, we obtained the highest Q-factor of 47.5, marked in red in Figure 7c. Additionally, the mode representation for the corresponding 16 kHz frequency is depicted in Figure 7d, showing that the antinode occurred at the end of the resonance cylinder.

#### 3.2.3. The Effect of Changing the Resonance Cylinder Position

**Figure 8:**
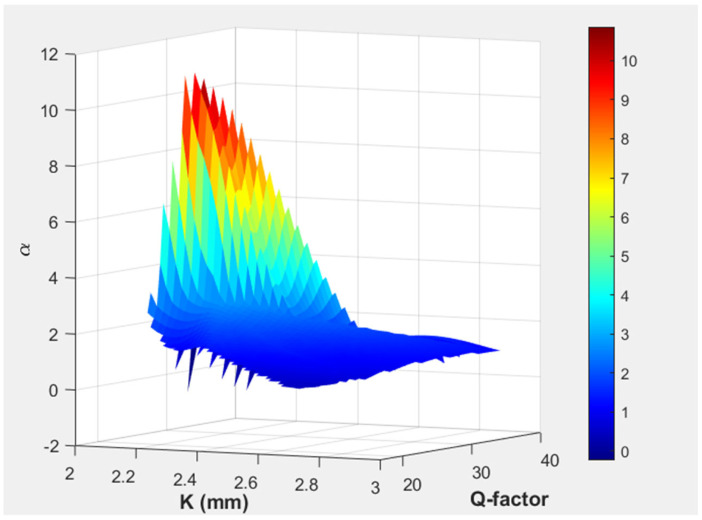
Variation in amplification factor α and Q-factor with respect to resonance cylinder position (*K*).

In Figure 8, the 3D surface plot demonstrates the variation in α and the Q-factor for the resonator cylinder position. The color gradient emphasizes areas of high and low pressure amplification, with peak values occurring at specific resonator positions where the Q-factor was optimized. This visualization aids in identifying the optimal placement of the resonance cylinder to achieve maximum pressure enhancement, offering insights into the resonance behavior and system efficiency. The maximum value of the Q-factor was observed in the initial position, while the maximum amplification was noted at a *K* of 2.09 mm.

#### 3.2.4. The Effect of Changing Both the Resonance and Cavity Cylinder Parameters

**Figure 9:**
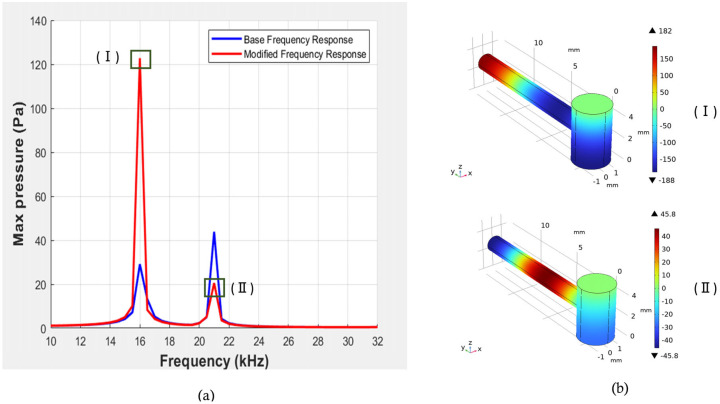
(**a**) Representation of pressure vs. frequency response for initial and modified parameters (R = 1.55 mm, L = 5.1 mm, r = 0.77 mm, l = 13 mm, K = 2.5 mm) of resonator. (**b**) Corresponding mode representations.

From Figure 9, we can observe that at 16 kHz, the maximum pressure value was 121.2 Pa, which was nearly 2.7 times higher than the previous maximum pressure value that was found with the initial parameters. Additionally, we achieved a Q-factor of 42 at 16 kHz, which was significantly greater than the previous Q-factor at this frequency. Also, mode representations for the corresponding frequencies are shown for a better understanding. 

**Figure 10:**
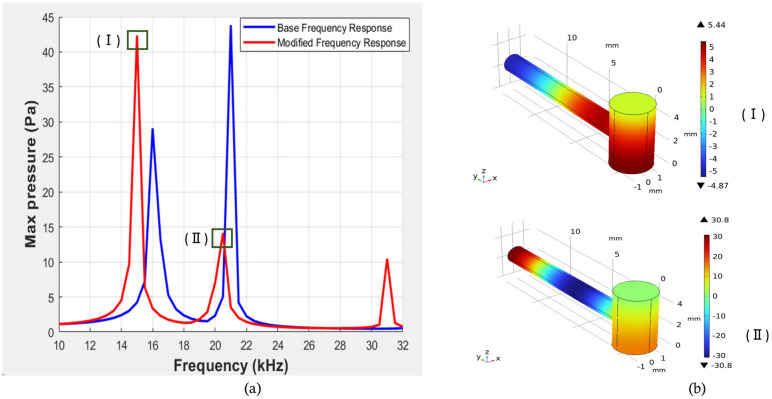
(**a**) Representation of pressure vs. frequency response for initial and modified parameters (R = 1.55 mm, L = 5.1 mm, r = 0.77 mm, l = 13 mm, K = 2.09 mm) of resonator. (**b**) Corresponding mode representations.

Figure 10 shows a comparison of the pressure vs. frequency response between the initial parameters and after the modification was applied to all the parameters. Here, we can observe that the maximum pressure value that we obtained was 42 Pa, which was less than the maximum pressure of the initial parameters. Additionally, mode representations for the corresponding frequency are shown in Figure 10b. An antinode can be observed in the outermost portion of the resonance cylinder in Figure 10bI, where the detection device will be connected, and in Figure 10bII, the antinode is present near the junction of the resonance and cavity cylinder.

This study focused on optimizing the design of a T-shaped PAR to enhance the sensitivity of noninvasive blood glucose detection using photoacoustic spectroscopy (PAS). We conducted computational simulations using COMSOL Multiphysics to systematically investigate how varying geometric parameters, such as the diameter and length of the cavity and resonance cylinders and the position of the resonant cylinder, affected the resonator’s performance. Our results showed that modifying these parameters significantly impacted the amplitude of the photoacoustic signal and the quality factor, both of which are critical for improving the detection sensitivity. A key finding was that a slight increase in the length and radius of the cavity cylinder led to a substantial increase in the amplification factor (α) to 12.8, which could be observed for the combination of parameters (b) mentioned in the table. This suggests that the length of the cavity cylinder is a crucial parameter for amplifying the photoacoustic signal. However, the Q-factor did not display a consistent trend regarding changes in the parameters, indicating that small deviations from the initial settings could significantly affect the resonator’s performance. This underscores the importance of precise alignment in practical applications. Additionally, we observed that increasing the diameter of the resonance cylinder from its base value amplified the sound pressure by a factor of 9.07, shown for combination (a) in Table 2. This suggests that there is an optimal diameter range for maximizing both the signal strength and quality. Overall, this study provides valuable insights into the geometric optimization of T-shaped resonators for PAS-based glucose detection. Finally, in combinations (c) and (d), as shown in Table 2, we mainly observed an effect on the amplification factor (α) and Q-factor when we combined the changes in all the parameters. Despite the promising simulation results, several environmental and biological factors could impact the real-world performance of the proposed PAS-based glucose sensing system. These factors include the ambient humidity, temperature fluctuations, and variability in human skin characteristics, such as the thickness, hydration level, and melanin content. Humidity can influence the absorption of mid-infrared light and acoustic propagation through air, while temperature variations may alter the thermoelastic response of the skin. Additionally, inter-individual differences in skin layers can affect the uniformity of optical absorption and acoustic signal generation. These factors must be carefully considered in future experimental studies. Potential mitigation strategies, including real-time environmental sensors and adaptive calibration algorithms, should be implemented for optimal detection. These findings indicate that the careful tuning of a resonator’s dimensions and positioning, along with the aforementioned factors, can significantly enhance signal amplification and detection sensitivity, paving the way for more accurate and reliable noninvasive glucose monitoring systems.

## 4. Conclusions

In conclusion, this study successfully demonstrated the potential of optimizing T-shaped PARs to enhance the sensitivity of noninvasive blood glucose detection using photoacoustic spectroscopy. By systematically varying geometric parameters such as the diameter, length, and position of the cavity and resonance cylinders, we identified key factors that influence both the amplitude of the photoacoustic signal and the quality factor. The results indicated that increasing the length and radius of the cavity cylinder significantly amplified the signal, while the diameter of the resonance cylinder also played a critical role in improving performance. These findings suggest that precise geometric optimization can lead to substantial improvements in the signal strength and detection sensitivity, which are essential for developing reliable noninvasive glucose monitoring devices. Future work should focus on further refining the resonator design to minimize losses and enhance the Q-factor, as well as on the experimental validation of the optimized resonator in real-world applications. This research contributes to the ongoing efforts to develop painless, accurate, and real-time glucose monitoring solutions for diabetic patients, ultimately improving their quality of life.

## Figures and Tables

**Table 1:** Summary of the parameters that were initially used for the resonator design and further analysis [31].

Structural Parameter	Symbol	Values (mm)
Position of the Resonance Cylinder	*K*	2.5
Radius of the Cavity Cylinder	*R*	1.5
Length of the Cavity Cylinder	*L*	5
Radius of the Resonance Cylinder	*r*	0.75
Length of the Resonance Cylinder	*l*	13

**Table 2:** Summary of the effect of changing the parameters on the amplification ratio and Q-factor for different combinations of PAR parameters.

	Parameters					Amplification Ratio (*α*)	Q-Factor
Combination	R (mm)	L (mm)	R (mm)	L (mm)	K (mm)		
a	1.5	5.0	0.77	13	2.5	9.07	47.5
b	1.55	5.1	0.75	13	2.5	12.76	47.5
c	1.55	5.1	0.77	13	2.5	2.7	42
d	1.5	5.0	0.75	13	2.09	11.6	29.42
e	1.55	5.1	0.77	13	2.09	0.93	42

## Data Availability

The original contributions presented in this study are included in the article. Further inquiries can be directed to the corresponding author.
